# Hydrostatic pressure suppresses fibrotic changes via Akt/GSK‐3 signaling in human cardiac fibroblasts

**DOI:** 10.14814/phy2.13687

**Published:** 2018-05-03

**Authors:** Ryo Tanaka, Masanari Umemura, Masatoshi Narikawa, Takayuki Fujita, Utako Yokoyama, Tomoaki Ishigami, Kazuo Kimura, Kouichi Tamura, Yoshihiro Ishikawa

**Affiliations:** ^1^ Cardiovascular Research Institute Yokohama City University School of Medicine Yokohama Japan; ^2^ Medical Science and Cardiorenal Medicine Yokohama City University School of Medicine Yokohama Japan; ^3^ Division of Cardiology Yokohama City University Medical Center Yokohama Japan

**Keywords:** Akt signaling, cardiac fibroblasts, hydrostatic pressure

## Abstract

Mechanical stresses play important roles in the process of constructing and modifying heart structure. It has been well established that stretch force acting on cardiac fibroblasts induces fibrosis. However, the effects of compressive force, that is*,* hydrostatic pressure (HP), have not been well elucidated. We thus evaluated the effects of HP using a pressure‐loading apparatus in human cardiac fibroblasts (HCFs) in vitro. In this study, high HP (200 mmHg) resulted in significant phosphorylation of Akt in HCFs. HP then greatly inhibited glycogen synthase kinase 3 (GSK‐3)*α*, which acts downstream of the PI3K/Akt pathway. Similarly, HP suppressed mRNA transcription of inflammatory cytokine‐6, collagen I and III, and matrix metalloproteinase 1, compared with an atmospheric pressure condition. Furthermore, HP inhibited collagen matrix production in a three‐dimensional HCF culture. Taken together, high HP suppressed the differentiation of fibroblasts into the myofibroblast phenotype. HP under certain conditions suppressed cardiac fibrosis via Akt/GSK‐3 signaling in HCFs. These results might help to elucidate the pathology of some types of heart disease.

## Introduction

Hemodynamic overload caused by hypertension or valvular heart disease, such as aortic valve stenosis, induces cardiac hypertrophy. Cardiac hypertrophy is an adaptive response to the acute and chronic hemodynamic overload in the heart. Cardiac hypertrophy causes diastolic dysfunction and harmful remodeling, eventually leading to heart failure and poor prognosis (van Heerebeek et al. 2006; Owan et al. 2006). Proliferation of cardiac fibroblasts and extracellular matrix (ECM) reorganization are observed in hypertrophied hearts (Biernacka and Frangogiannis 2011). Cardiac fibroblasts account for 60%–70% of all cells in the heart and play a central role in ECM protein synthesis, which is a critical cause of ventricular stiffness as well as impairment of left ventricular diastolic function (Krenning et al. 2010; Biernacka and Frangogiannis 2011).

Mechanical stimulation and humoral factors, such as angiotensin and catecholamine, may contribute to not only enlargement of cardiac myocytes, but also to activation and proliferation of cardiac fibroblasts, and differentiation of cardiac fibroblasts into myofibroblasts, resulting in cardiac hypertrophy, excessive accumulation of ECM, and fibrosis (Biernacka and Frangogiannis 2011). Myofibroblasts express *α*‐smooth muscle actin (*α*‐SMA), which is associated with increased contractile force (Hinz et al. 2001).

It has previously been reported that stretch stress promotes differentiation of fibroblasts into the myofibroblast phenotype, resulting in increased synthesis of ECM, such as fibronectin and collagen (Wang et al. 2003; Sarrazy et al. 2014). In contrast, little information is available regarding the effects of compressive force, that is*,* hydrostatic pressure (HP), on cell physiology (Sakai et al. 2007). Some studies have suggested that HP, as an external stimulus, plays an important role in the differentiation of fibroblasts into the myofibroblast phenotype and in ECM protein synthesis in human cardiac fibroblasts (HCFs). Zhao et al. (2015) demonstrated that HP is an active regulator for bone marrow mesenchymal stem cells (BMSCs). They also suggested that the cytoskeletal regulatory proteins Ras homolog gene family member A (RhoA) and Ras‐related C3 botulinum toxin substrate 1 (Rac1) are critical to pressure‐induced proliferation and differentiation, stress fiber assembly, and mitogen‐activated protein kinase (MAPK) activation in BMSCs. Xie et al. (2014) reported that the APJ receptor (angiotensin II receptor‐like 1) acts as a sensor in HP‐induced cardiomyocyte hypertrophy via the PI3K‐Akt pathway and autophagy. They showed that HP (180 mmHg) increases the expression of APJ, which activates the PI3K pathway, induces cell autophagy, and stimulates H9C2 cell hypertrophy.

We previously reported that arterial media‐mimetic constructs, consisting of either human umbilical arterial smooth muscle cells (hHASMCs) or rat neonatal aortic smooth muscle cells (SMCs) were grown in a multilayer cell culture (Ishiwata et al. 2014). Furthermore, we reported that extremely high HP during repeated cell seeding resulted in stronger arterial grafts with an elastic layer structure from cultured human SMCs than the arterial grafts which we had previously constructed under atmospheric pressure (AP) conditions (Yokoyama et al. 2017). Our grafts were highly elastic with abundantly formed elastic fibers, and the patch graft could be sutured to rat aorta. Our data demonstrated that HP beyond physiological conditions promoted stress fiber formation and fibrillogenesis via the Rho/ROCK pathway and three‐dimensional assembly of SMCs.

However, little is known about how HP is converted into intracellular signals that stimulate the differentiation of cardiac fibroblasts into myoblasts, and how HP regulates *α*‐SMA expression in HCFs. In this study, we show that excessive HP inhibits collagen matrix production and suppresses the differentiation of fibroblasts into the myofibroblast phenotype, resulting in the prevention of cardiac fibrotic changes via Akt/glycogen synthase kinase 3 (GSK‐3) signaling in HCFs.

## Methods

### Reagents

Phospho‐Akt (Ser473 and Thr308) antibody, phospho‐GSK‐3*α* (Ser21)/GSK‐3*β* (Ser9) antibody, phospho‐p38 (Thr180/Tyr182) antibody, Akt antibody, GSK‐3*α*/*β* antibody, p38 antibody, and glyceraldehyde‐3‐phosphate dehydrogenase (GAPDH) antibody were purchased from Cell Signaling (Danvers, MA, USA). *α*‐SMA antibody was purchased from Sigma‐Aldrich (St. Louis, MO, USA). Alexa Fluor 488 goat anti‐mouse immunoglobulin G (IgG) and 4′,6‐diamidino‐2‐phenylindole (DAPI) were purchased from Invitrogen (Carlsbad, CA, USA). ML221 was purchased from Sigma‐Aldrich. LY294002 was purchased from Cell Signaling.

### Cell culture

Human Cardiac Fibroblasts‐adult cells (No 6330) (HCFs) were purchased from ScienCell Research Laboratories (Carlsbad, CA, USA). HCFs were cultured in fibroblast medium‐2 (FM‐2; Cat. No.2331, ScienCell Research Laboratories), a manufacturer fibroblast medium supplemented with 1% penicillin/streptomycin, 1% fibroblast growth supplement‐2 (FGS‐2), and 2% fetal bovine serum (FBS) (Heublein et al. 2007). All cells were maintained in a humidified atmosphere of 95% air and 5% CO_2_ at 37°C. The 4th through 8th passages of HCFs were used for the experiments. HCFs at AP were used as controls.

### HP‐loading apparatus

**Figure 1:**
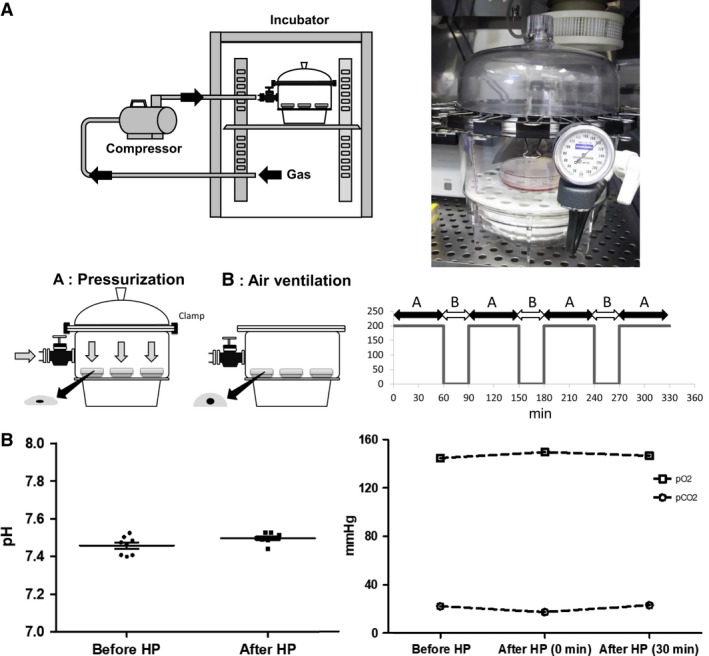
(A–B) Pressure loading apparatus and pressurizing method. Ventilation time of 30 min every 60 min of pressurization minimized the pH and dissolved O_2_ and CO_2_ in the culture medium.

HP was applied to the HCFs using an apparatus that combined a desiccator (Cat.No.VDR‐20/1‐1462‐01, As One, Osaka, Japan) and manometer (Fig. 1), as previously reported (Tomasek et al. 2002; Okada et al. 2008). Gas from an incubator (37°C, 5% CO_2_) was pumped into the desiccator and pressurized. Gas leakage was completely prevented with a clamp using several double clips. The pressure levels inside the apparatus were monitored with the manometer, and the internal temperature levels were monitored with a thermometer. Ph, dissolved O_2_, and CO_2_ levels in the culture medium were checked with i‐STAT 1 analyzer (Abbot, Lake Forest, IL, USA). The ventilation time was set for 30 min every 60 min of pressurization to avoid changing the experimental conditions. The composition of the dissolved gas was not changed in this protocol (Fig. 1).

### Phosphokinase antibody array

The effects of HP on protein phosphorylation in HCFs were comprehensively evaluated using a phosphokinase antibody array, as previously reported (Umemura et al. 2014). HCFs were maintained at 0 mmHg (AP) or 200 mmHg (HP conditions) for 60 min. Microarray experiments were carried out using the PathScan Signaling Antibody Array Kit (Cell Signaling) according to the manufacturer's instructions. Signal intensities were quantified using ATTO CS Analyzer 4 software (ATTO, Tokyo, Japan).

### Western blotting

Western blot analyses were performed as previously described (Umemura et al. 2014; Oda et al. 2017). The following primary antibodies were used for immunoblotting: phospho‐Akt (Ser473) antibody (1:1000), phospho‐GSK‐3*α* (Ser21), phospho‐GSK‐3*β* (Ser9) antibody (1:1000), phospho‐p38 (Thr180/Tyr182) antibody (1:1000), Akt antibody (1:1000), GSK‐3*α*/*β* antibody (1:1000), p38 antibody (1:1000), and *α*‐SMA antibody (1:5000). GAPDH antibodies (1:5000) were used as loading controls to normalize the data. Chemiluminescence detection was performed using the Pierce ECL reagent (ThermoFisher, Waltham, MA, USA). Signal intensities of the bands were quantified using ATTO CS Analyzer 4 software (ATTO).

### Quantitative real‐time reverse transcriptase– polymerase chain reaction

Total RNA from HCFs was extracted using the RNAiso Plus reagent (Takara Bio, Shiga, Japan), and reverse transcription reactions were performed using the PrimeScript RT reagent kit (Takara Bio), as previously described (Narikawa et al. 2018). Quantitative polymerase chain reaction (PCR) was prepared using SYBR Fast qPCR Mix (Takara Bio). Reverse transcription–polymerase chain reaction (RT–PCR) was performed on the StepOnePlus Real‐Time PCR System (Applied Biosystems, Foster City, CA, USA). PCR consisted of an initial cycle of 95°C for 30 sec, then 40 cycles, each consisting of denaturation at 95°C for 5 sec, followed by annealing and primer extension at 60°C for 30 sec. The 2^−ΔΔCT^ method was used to determine relative gene expression levels, using GAPDH to normalize the data. The sequences of the specific primers were as follows: ACTA2 (forward, 5′‐ ATTGCCGACCGAATGCAGA ‐3′; reverse, 5′‐ ATGGAGCCACCGATCCAGAC ‐3′), col1A1 (forward, 5′‐ CCCGGGTTTCAGAGACAACTTC ‐3′; reverse, 5′‐ TCCACATGCTTTATTCCAGCAATC ‐3′), col3A3 (forward, 5′‐ CTTCTCTCCAGCCGAGCTTC ‐3′; reverse, 5′‐ TGGAGGTTAGTGGGAGCATC ‐3′), MMP‐1 (forward, 5′‐ ACATGAGTCTTTGCCGGAGG ‐3′; reverse, 5′‐ AACAAGGTTGACTTTATTCCAAACA ‐3′), IL‐6 (forward, 5′‐ AGTTCCTGCAGAAAAAGGCAAAG ‐3′; reverse, 5′‐ CATTTGCCGAAGAGCCCTCA ‐3′), TNF‐*α* (forward, 5′‐ CACTGAAAGCATGATCCGGG ‐3′; reverse, 5′‐ CTGGGGAACTCTTCCCTCTGG ‐3′), and GAPDH (forward, 5′‐CCCATCACCATCTTCCAGGAGCG‐3′; reverse, 5′‐GGCAGGGATGATGTTCTGGAGAGCC‐3′) (Sádaba et al. 2016).

### Immunofluorescence staining

Immunofluorescence staining was performed as previously described (Sato et al. 2016; Ohtake et al. 2017). Briefly, cells were grown to 80% confluence on 12‐mm coverslips, washed with phosphate‐buffered saline (PBS), and fixed with 2% formaldehyde for 15 min. The coverslips were preincubated for 30 min at room temperature in 0.01% Triton X‐100 (PBS‐Triton), then the cells were treated with primary antibody (*α*‐SMA antibody, 1:500) overnight at 4°C, and thereafter with Alexa Fluor 488 goat anti‐mouse IgG (1:1000) as the secondary antibody for 1 h at room temperature. Cells were stained with DAPI (1:5000) to detect cell nuclei. Cells were then visualized using fluorescence microscopy with an inverted microscope (Nikon, Tokyo, Japan).

### Collagen gel contraction assay

Following culture for 24 h at AP and 16 h at HP of 200 mmHg (total 24 h, including ventilation time), HCFs were trypsinized and seeded into 24‐well plates at a density of 2 × 10^5^ cells/ml in collagen gel. Following 24‐h incubation, the surface area of the collagen gel was evaluated according to the manufacturer's instructions (Tian et al. 2002; Zhang et al. 2011). This assay was performed using the CytoSelect 24‐Well Cell Contraction Assay Kit (Cell Biolabs, San Diego, CA, USA). The surface area of the collagen gel was quantified using ATTO CS Analyzer 4 software (ATTO).

### Cell viability assay

The XTT Cell Proliferation Kit (ATCC, Manassas, VA, USA) was used to analyze cell viability, as previously reported (Kim et al. 2017; Umemura et al. 2017).

### Data analysis and statistics

Statistical comparisons between groups were performed using Student's *t*‐test or one‐factor analysis of variance (ANOVA) with the Tukey post hoc test. The criterion of statistical significance was set at *P *<* *0.05.

## Results

### HP resulted in phosphorylation of 11 proteins including Akt (Ser473) in HCFs

We examined the effects of HP compared to AP conditions in cultured HCFs. We performed a phosphokinase antibody array to investigate the effect of HP on phosphorylation in HCFs when exposed to pressure of 200 mmHg for 60 min. The experimental system was composed of a pressure vessel containing a cell culture dish, and a compressor for supplying the pressurized air to the pressure vessel (Fig. 1A). The compressor was supplied with moist air kept at 37°C and appropriate CO_2_ concentration (Fig. 1B). To adjust the pH to 7.4 in the cell culture medium, we changed the CO_2_ concentration from 3.6% to 5% in the supplied air. The pressure vessel was also kept at 37°C.

**Figure 2:**
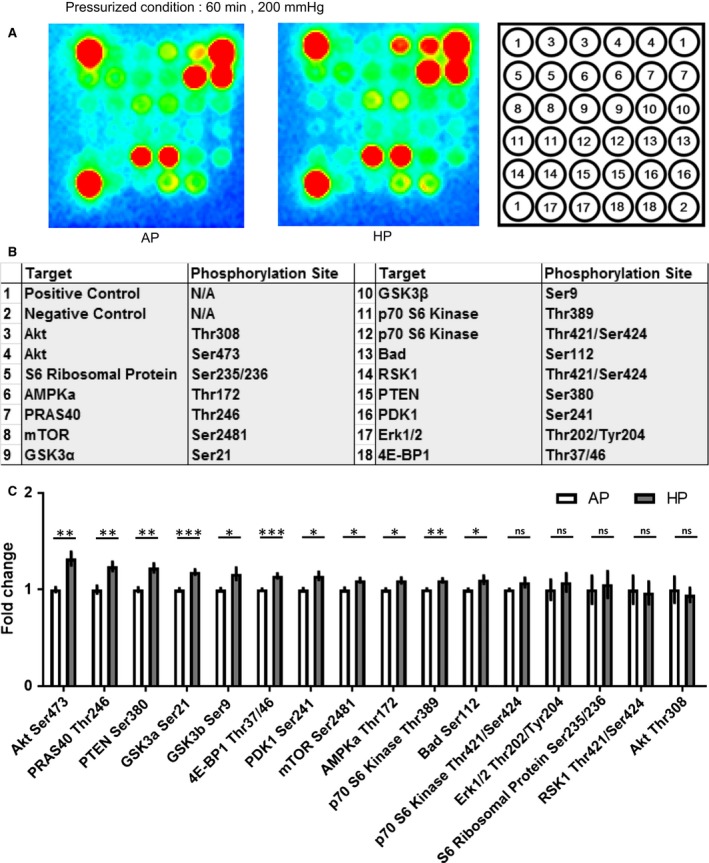
Phosphorylated antibody array showed that hydrostatic pressure resulted in phosphorylation of several proteins. (A) Phosphorylated antibody map with atmospheric pressure (AP) and hydrostatic pressure (HP). The left map shows phosphorylated proteins in the AP group. The right map shows phosphorylated proteins in the HP group (200 mmHg, 60 min). (B) List of target proteins and phosphorylation sites in phosphorylated antibody array. (C) Histograms of protein phosphorylation under AP conditions (*white*) and HP conditions (200 mmHg; *gray*) for 60 min. HP resulted in significant phosphorylation of several proteins including Akt (Ser473); unpaired *t*‐test, *n* = 4, * *P *<* *0.05, ** *P < *0.01, *** *P < *0.001, ns: no significant difference).

Comparison of phosphorylation levels in 18 proteins (including one positive and one negative control) with/without HP for 60 min showed that HP significantly increased phosphorylation of 11 proteins, including serine/threonine kinase Akt (also known as protein kinase B [PKB]; Ser473; Fig. 2) (Bakin et al. 2000). It is well‐known that the PI3K/Akt pathway is one of the main pathways in the cell signaling transduction involved in cardiac hypertrophy (Ackermann et al. 2017). One study reported that HP (180 mmHg) activated the PI3K pathway in H9C2 cardiomyocytes (Xie et al. 2014). Our results were in accord with this result. Furthermore, our data suggested that HP also affects Akt signaling in HCFs. Therefore, we focused on the phosphorylation of Akt (Ser473) among the proteins we evaluated, because phosphorylation of Akt, which is involved in cardiac fibrotic changes, was highly increased in our study (Villarreal et al. 2009; Guo et al. 2016; Phosri et al. 2017).

### HP promoted phosphorylation of Akt (Ser473) and GSK‐3α and decreased that of p38 in HCFs

**Figure 3:**
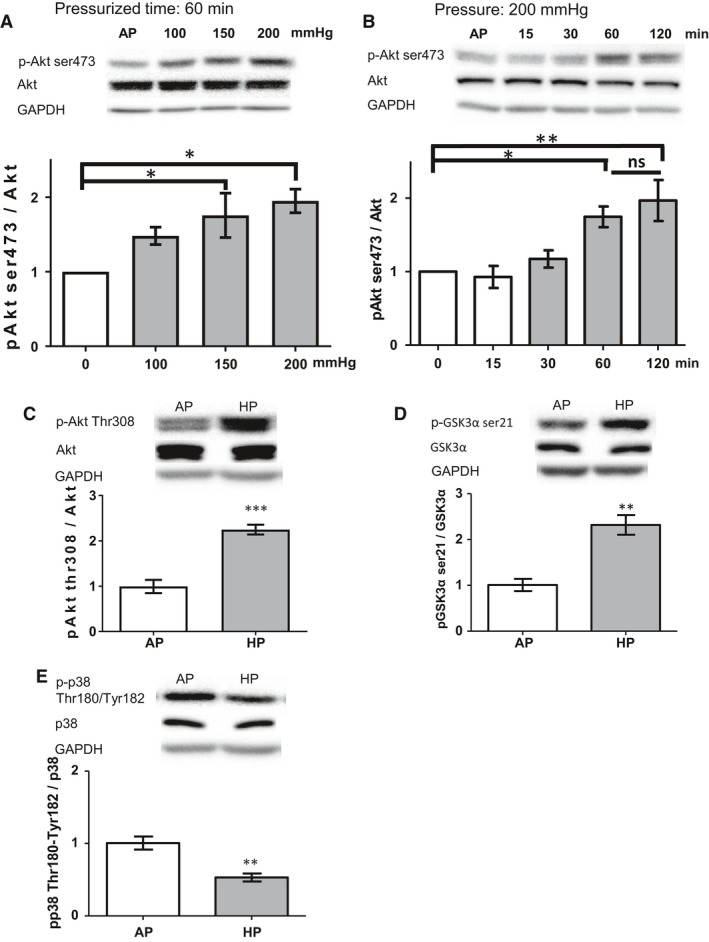
HP resulted in phosphorylation of Akt (Ser473) in a time‐ and pressure‐dependent manner and enhanced or attenuated the phosphorylation of several kinases and transcription factors. (A) HP for 60 min resulted in the phosphorylation of Akt in a pressure‐dependent manner (one‐way ANOVA with Tukey post hoc test, *n* = 4, * *P *<* *0.05). (B) HP resulted in the phosphorylation of Akt in a time‐dependent manner when the pressure was fixed at 200 mmHg (one‐way ANOVA with Tukey post hoc test, *n* = 4, * *P *<* *0.05, ** *P *<* *0.01, ns: no significant difference). (C–E) HP (200 mmHg, 60 min) resulted in phosphorylation of glycogen synthase kinase (GSK)‐3*α* (Ser21) and in dephosphorylation of p38 (Thr180/Tyr182); unpaired *t*‐test, *n* = 4, * *P *<* *0.05, ** *P *<* *0.01, *** *P *<* *0.001).

To investigate the conditions under which HP promotes phosphorylation of Akt in the PI3K/Akt pathway in HCFs, we first investigated the effect of HP on the phosphorylation of Akt in HCFs when exposed to different pressure levels (AP, 100, 150, and 200 mmHg) for 60 min. Our results indicated that Akt phosphorylation in HCFs increased the most at 200 mmHg (Fig. 3A). We next evaluated the time‐course effect of high HP on the phosphorylation of Akt in HCFs when exposed to pressure of 200 mmHg for 15, 30, 60, or 120 min. We found that HP increased phosphorylation of Akt (Ser473) in a time‐dependent manner. High HP (200 mmHg) resulted in the greatest increase in Akt phosphorylation in HCFs after 60 min (Fig. 3B).

Taken together, our results indicated that phosphorylation of Akt increased the most when HCFs were exposed to pressure of 200 mmHg for 60 min. Therefore, we evaluated the phosphorylation of proteins related to the PI3K/Akt pathway under this pressure condition (200 mmHg).

GSK‐3*α* data were consistent with the results from our phosphokinase antibody array, except for Akt (Thr308) (Fig. 3C and D). GSK‐3 is a ubiquitously expressed, serine/threonine kinase known for its role in regulating glycogen synthase (Lal et al. 2015). It is well‐known that GSK‐3 acts downstream of Akt, and phosphorylation of GSK‐3 inactivates its function, thereby regulating cardiac function and construction. We also investigated the phosphorylation of p38 MAPK in the transduction pathway. p38 is involved in inducing the expression of proinflammatory cytokines, extracellular matrix metalloproteinases (MMPs), and collagen in HCFs (Sinfield et al. 2013; Jiang et al. 2016). p38 MAPK is one of the most important factors regulating cardiac function. In our study, HP suppressed phosphorylation of p38 (Fig. 3E). Our findings confirmed that HP increased the phosphorylation of the PIK3/Akt/GSK‐3 pathway and the dephosphorylation of p38 MAPK in HCFs.

On the basis of these results, we hypothesized that HP inhibited the inflammatory reaction, the production of ECM proteins, and MMPs in HCFs.

### HP inhibited the transcription of ECM, inflammatory cytokines, and MMPs in HCFs

**Figure 4:**
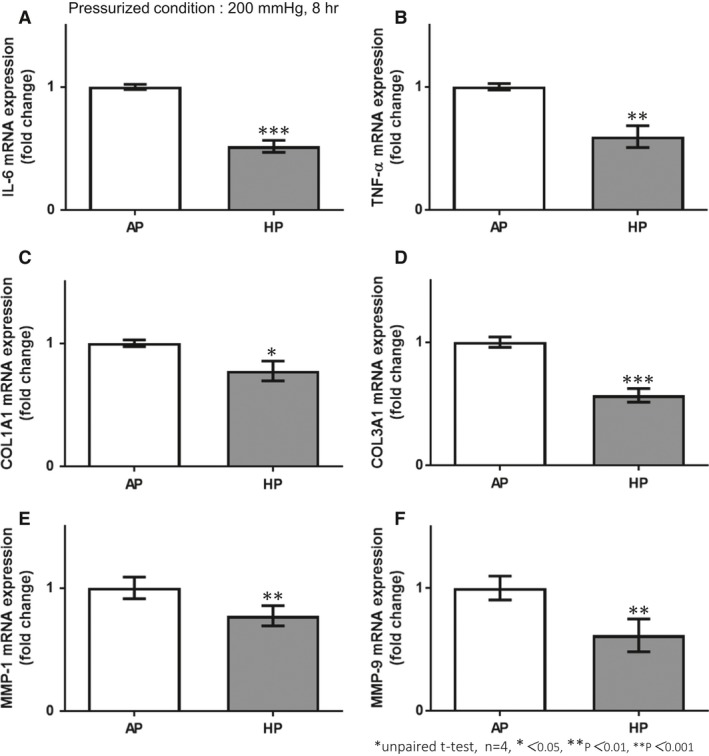
HP resulted in suppression of mRNA expression related to tissue healing factors, such as extracellular matrix, matrix metalloproteinases, and inflammatory cytokines in human cardiac fibroblasts. HP (200 mmHg, 8 h) inhibited mRNA expressions of COL1A1, CO3A1, matrix metalloproteinase (MMP)‐1, MMP‐9, interleukin (IL)‐6, and tumor necrosis factor alpha (TNF‐*α*) in human cardiac fibroblasts (HCFs); unpaired *t*‐test, *n* = 4, * *P *<* *0.05, ** *P *<* *0.01, *** *P *<* *0.001).

It has been established that mechanical stress, for example*,* stretch force, promotes the differentiation from fibroblasts into the myofibroblast phenotype, and elevates fibronectin protein levels and the production of ECM proteins, such as collagen (Wang et al. 2003; Guo et al. 2013; Sarrazy et al. 2014). In this study, we investigated the effect of HP on ECM proteins, inflammatory cytokines, and MMPs, which play important roles in cardiac repair or chronic cardiac overload with myocardial damage. High HP markedly inhibited mRNA expression of interleukin 6 (IL‐6), tumor necrosis factor alpha (TNF‐*α*), collagen type 1 alpha 1 (COL1A1), which encodes collagen I, collagen type 3 alpha 1 (COL3A1), which encodes collagen III, MMP‐1, and MMP‐9 in HCFs (Fig. 4A–F). High HP did not change mRNA expression of fibronectin and transforming growth factor beta (TGF‐*β*) (data not shown). These results suggested that high HP suppressed the tissue healing response in HCFs.

### HP inhibited differentiation and contractile function of cardiac myoblasts

**Figure 5:**
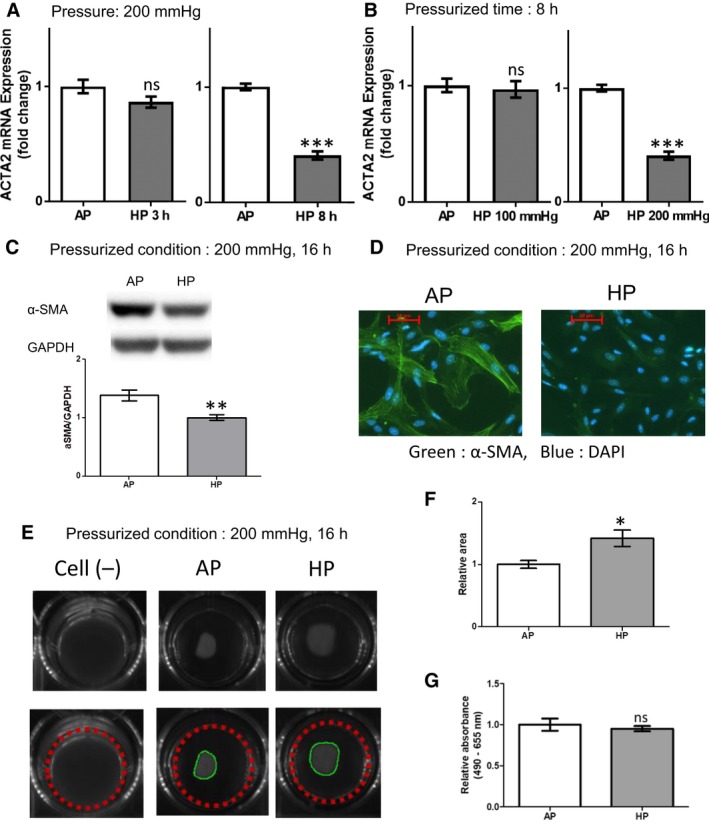
HP inhibited differentiation of fibroblasts into myofibroblasts and inhibited contractile function in HCFs. (A) Quantitative mRNA expression of alpha‐actin‐2 (ACTA2) after 3 h of HP (*white*) and 8 h of HP (*gray*); unpaired *t*‐test, *n* = 4, *** *P *<* *0.001, ns: no significant difference). HP inhibited mRNA expression of ACTA2 after 8 h of HP, but not after 3 h. (B) Quantitative mRNA expression of ACTA2 after 8 h of AP (*white*) or HP (*gray*); unpaired *t*‐test, *n* = 4, *** *P *<* *0.001, ns: no significant difference). HP of 200 mmHg inhibited mRNA expression of ACTA2, but not HP of 100 mmHg. (C) Representative images of western blot (*upper*) and normalized protein expression of *α*‐smooth muscle actin (*α*‐SMA) at 16 h determined by densitometry (*lower*); unpaired *t*‐test, *n* = 4, ** *P *<* *0.01). (D) Representative immunocytochemistry of *α*‐SMA in HCFs (DAPI, 4′,6‐diamidino‐2‐phenylindole); Magnification = × 40; bar = 50 *μ*m. (E) Effects of AP and HP on the ability of HCF cells to contract floating collagen gels. HP for 16 h inhibited contraction of the collagen matrix in three‐dimensional HCF culture. Representative photographs of floating gels (E) and relative area of collagen matrix (AP and HP; (F)); unpaired *t*‐test, *n* = 4, * *P *<* *0.05). (G) HP for 16 h did not affect cell proliferation (unpaired *t*‐test, *n* = 4, ns: no significant difference).

Differentiation from fibroblasts into the myofibroblast phenotype is characterized by expression of *α*‐SMA, which is induced by TGF‐*β* 1, cytokines, ECM components, and various growth factors (Souders et al. 2009). In myofibroblasts, *α*‐SMA is one of the most important contractile proteins required for collagen remodeling (Arora and McCulloch 1994). Therefore, we examined the effect of HP on *α*‐SMA expression in HCFs. High HP for 8 h markedly decreased mRNA expression of alpha‐actin‐2 (ACTA2), which encodes *α*‐SMA, not for 3 h (Fig. 5A). High HP of 200 mmHg for 8 h markedly decreased mRNA expression of alpha‐actin‐2 (ACTA2), not that of 100 mmHg (Fig. 5B). High HP also decreased protein expression of *α*‐SMA in HCFs (Fig. 5C). In accordance with the western blotting data, immunofluorescence images showed that high HP decreased the expression of *α*‐SMA stress fibers (Fig. 5D). Furthermore, high HP of 200 mmHg for 16 h inhibited collagen matrix production compared to AP in a three‐dimensional culture of HCFs (Fig. 5E and F). These data indicated that high HP decreased contractility in cultured HCFs.

Taken together, the data showed that high HP resulted in phosphorylation of Akt and inactivation of GSK‐3 by Akt‐induced GSK‐3 phosphorylation, resulting in suppressed *α*‐SMA expression.

### HP controlled cell response by acting on the apelin receptor

**Figure 6:**
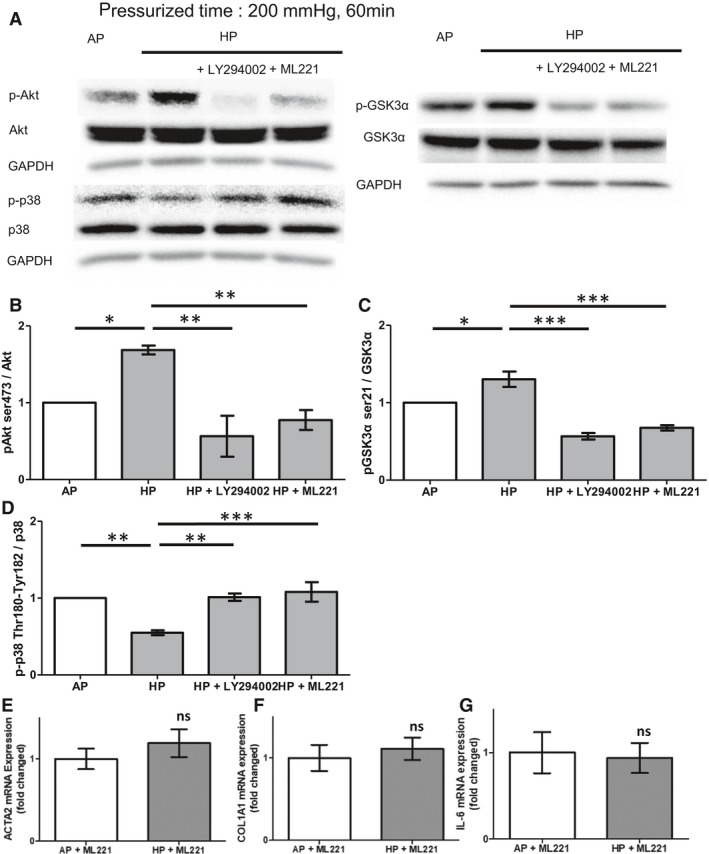
Apelin receptor antagonist suppressed HP effects. (A) Representative images of western blot analysis of pAkt, GSK‐3*α* and p38 in the presence of LY294002 or ML221. (B–G) Both ML221 (50 *μ*mol/L), an apelin receptor antagonist, and LY294002 (10 *μ*mol/L), a PI3K‐Akt inhibitor, suppressed HP‐induced phosphorylation of Akt (B) and GSK‐3*α* (C). (one‐way ANOVA with Tukey post hoc test, *n* = 4, * *P *<* *0.05, ** *P *<* *0.01, *** *P *<* *0.001). ML221 did not suppress HP‐induced dephosphorylation of p38 (D). ML221 (50 *μ*mol/L) suppressed HP‐induced mRNA transcription of ACTA2 (E), COL1A1 (F), and IL‐6 (G); unpaired *t*‐test, *n* = 4, ns: no significant difference).

To investigate the upstream processes of Akt and the receptor of HP in HCFs, we focused on the apelin receptor, also known as the APJ receptor (angiotensin II receptor‐like 1), a Gi/o protein‐coupled receptor which binds the endogenous ligand apelin. The apelin/APJ system plays an important role in the maintenance of heart development and cardiovascular homeostasis (Bakin et al. 2000). APJ is an HP‐sensitive receptor involved in cardiomyocyte (H9C2) hypertrophy via the autophagy via PI3K‐Akt pathway (Xie et al. 2014). It has been reported that APJ knockout mice are protected from hypertrophy after transaortic constriction (Scimia et al. 2012). Therefore, we hypothesized that APJ is a promising sensor in the response of HCFs to HP. We thus examined whether Akt inhibitor (LY294002) and APJ receptor antagonist (ML221) had inverse effects to HP on the phosphorylation of Akt, GSK‐3*α* and p38 MAPK. LY294002 and ML221 negated HP‐induced phosphorylation of Akt (Ser473), and GSK‐3*α* in HCFs (Fig. 6A, B and C). Similarly, both LY294002 and ML221 negated HP‐induced dephosphorylation of p38 (Fig. 6A and D). Based on these data, APJ may act as an HP sensor upstream of Akt signaling. Conversely, both GSK‐3 and p38 may act downstream of Akt signaling. Furthermore, ML221 inhibited HP‐induced mRNA expression of ACTA2, COL1A1, and IL‐6, suggesting that these genes are regulated via the APJ receptor (Fig. 6E, F and G).

## Discussion

**Figure 7:**
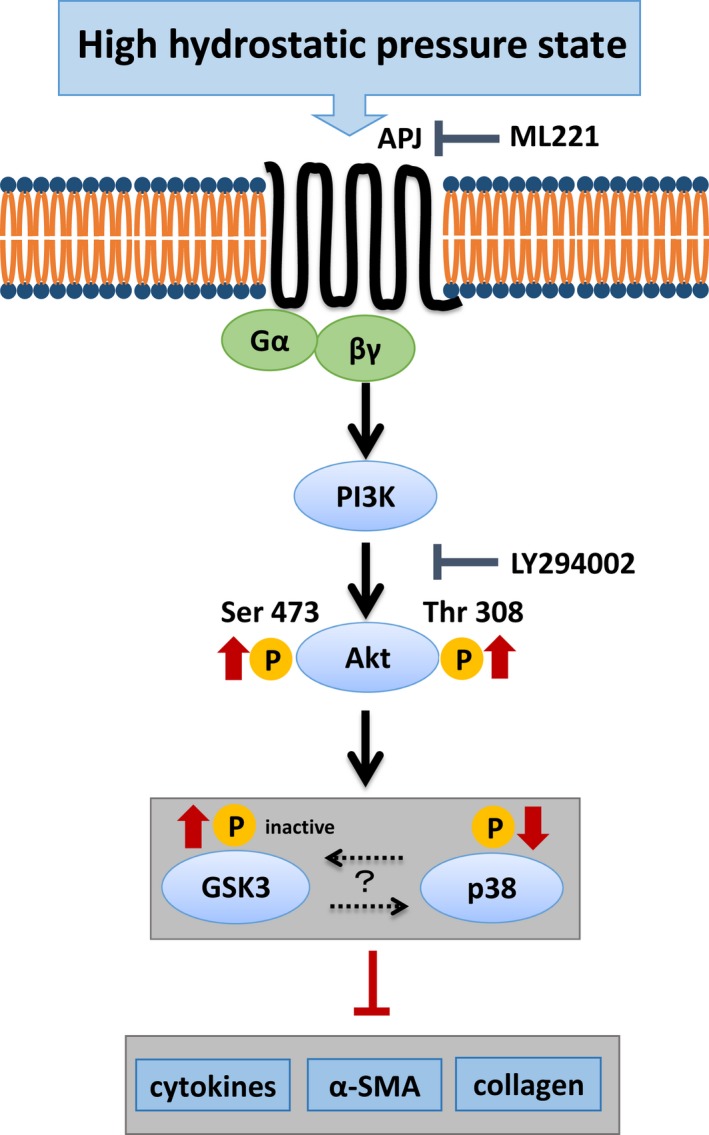
Proposed mechanism of effects of HP on HCFs. HP acts on apelin receptor to suppress the tissue healing response via the PI3K/Akt pathway.

In this study, we investigated the effects of internal pressure, that is, HP, in culture human cardiac fibroblast cells. Although many reports have explored the role of mechanical tension, for example*,* stretch force, this is the first report on the response of cardiac fibroblasts to HP. Our results indicated that HP inhibits differentiation of HCFs into the myofibroblast phenotype, and decreases gene expression of ECM proteins, inflammatory cytokines, and MMPs. A proposed signaling schema of HP‐induced PI3K/Akt activation is shown in Figure 7. The mechanism of the effect of internal pressure on the organization of heart structure may be different from that of acute or chronic hemodynamic overload and stretch activation. When cardiac tissue is injured, successful repair depends on the strength of the intracellular matrix and the degree of scar reduction due to myofibroblast contraction. Therefore, excessively high internal pressure represents a disadvantage with regard to tissue repair.

This study had several limitations. First, our study was performed using a custom‐made pressure incubator based on previous reports. This custom‐made HP loading apparatus applied HP using mixed gas in an airtight container. Continuous HP could only be applied for short time periods in this study because ventilation was required every 60 min of pressurization so that the experimental conditions would not be altered. Second, we used continuous HP in this study, and we did not further investigate the effect of periodic HP to mimic heart rhythm. Therefore, further investigation may be necessary using a newly developed HP system that allows observation for longer time periods and that is capable of producing periodic HP to mimic heart rhythm.

We found that the APJ receptor antagonist ML221 had inverse effects to HP on the phosphorylation of Akt, GSK‐3*α,* and p38 MAPK in HCFs. This finding has an important implication. Apelin and APJ are endogenous counter‐regulators of the renin–angiotensin–aldosterone system (RAAS). APJ is also a powerful candidate sensor in the response to HP in HCFs. It has been reported that excessive HP increased angiotensin‐II levels in mesangial and podocyte cells via angiotensin receptor 1 (AT1), resulting in apoptosis and detachment of nonviable apoptotic podocytes (Abu Hamad et al. 2017). AT1 receptor is responsible for most of the pathophysiological actions of angiotensin II. APJ and AT1 receptors are coexpressed in cardiovascular tissues (Iwanaga et al. 2006; Chun et al. 2008). Taken together, AT1 receptor may also be a candidate sensor in the response to HP in HCFs. Further investigation is needed to find HP targets beyond APJ in HCFs.

The molecular mechanisms of the HP‐induced phenotype change from fibroblast to the myofibroblast phenotype in HCFs, such as ECM, IL‐6, TNF‐*α*, and collagen I production, are not fully understood. Here, we demonstrated that excessive HP suppresses the differentiation of fibroblasts into the myofibroblast phenotype, which is characterized by expression of *α*‐SMA via the PI3K/Akt/GSK‐3 pathway. As a result, the proper tissue healing response might not be promoted. We propose that decreasing excessive hemodynamic overload is key to inducing adequate tissue repair of cardiac fibroblasts in myocardial hypertrophy.

## Conflict of Interest

The authors disclose no conflicts of interest.
